# Oxidation-Specific Epitopes (OSEs) Dominate the B Cell Response in Murine Polymicrobial Sepsis

**DOI:** 10.3389/fimmu.2020.01570

**Published:** 2020-07-31

**Authors:** Oliver Nicolai, Christian Pötschke, Dina Raafat, Julia van der Linde, Sandra Quosdorf, Anna Laqua, Claus-Dieter Heidecke, Claudia Berek, Murthy N. Darisipudi, Christoph J. Binder, Barbara M. Bröker

**Affiliations:** ^1^Department of Immunology, Institute of Immunology and Transfusion Medicine, University Medicine Greifswald, Greifswald, Germany; ^2^Department of Microbiology and Immunology, Faculty of Pharmacy, Alexandria University, Alexandria, Egypt; ^3^Department of General Surgery, Visceral, Thoracic and Vascular Surgery, University Medicine Greifswald, Greifswald, Germany; ^4^German Rheumatism Research Centre (DRFZ), Berlin, Germany; ^5^Department of Laboratory Medicine, Medical University of Vienna, Center for Molecular Medicine of the Austrian Academy of Sciences, Vienna, Austria

**Keywords:** CASP, polymicrobial sepsis, B cell response, polyreactive antibodies, oxidation-specific epitopes

## Abstract

In murine abdominal sepsis by colon ascendens stent peritonitis (CASP), a strong increase in serum IgM and IgG antibodies was observed, which reached maximum values 14 days following sepsis induction. The specificity of this antibody response was studied in serum and at the single cell level using a broad panel of bacterial, sepsis-unrelated as well as self-antigens. Whereas an antibacterial IgM/IgG response was rarely observed, studies at the single-cell level revealed that IgM antibodies, in particular, were largely polyreactive. Interestingly, at least 16% of the IgM mAbs and 20% of the IgG mAbs derived from post-septic mice showed specificity for oxidation-specific epitopes (OSEs), which are known targets of the innate/adaptive immune response. This identifies those self-antigens as the main target of B cell responses in sepsis.

## Introduction

Sepsis, by definition, is a life-threatening organ dysfunction caused by a dysregulated host response to infection (1, 2), which is associated with high morbidity and mortality (3, 4). Due to an aging population, a steady increase in surgical interventions, and the occurrence of antibiotic resistance, sepsis is still of high clinical relevance (4–6).

B cells have been ascribed a protective function in sepsis which encompasses antibody-dependent as well as -independent mechanisms (7, 8). However, during sepsis, a large number of B cells and other immune cells are lost by apoptosis (9, 10) and it is assumed that B cell responses are severely impaired (11, 12). In fact, Mohr et al. have shown that B cell priming with defined antigens is defective in sepsis (13). By contrast, we and other research groups have shown that sepsis induces high concentrations of serum IgM and IgG antibodies of unknown specificities (13, 14). Since these may be responsible for the observed antibody-mediated protection, we set out to examine their antigen specificities in a mouse model of abdominal sepsis.

During sepsis, the organism is flooded with bacterial antigens as well as self-antigens, which are released by dying host cells. Moreover, there is an abundance of danger signals, both pathogen- and damage-associated molecular patterns (PAMPs and DAMPs, respectively) (15, 16). These may, on the one hand, act as adjuvants in an antigen-driven B cell response and, on the other, trigger a polyclonal B cell reaction (17–19). In addition to these danger signals, inflammation and cell death are accompanied by lipid peroxidation, resulting in the generation of oxidation-specific epitopes (OSEs), which are also recognized by pattern recognition receptors (PRR) of the innate immune system exerting an adjuvant effect (20–22). All of these factors could contribute to the B cell response in sepsis.

During sepsis, antibody production is induced by T cell-dependent (TD) as well as -independent (TI) mechanisms (14). In a TD immune reaction, follicular B cells are activated via the B cell receptor. With the help of activated T cells, they differentiate and form germinal centers, where class switch to all Ig (sub)classes and somatic hypermutation take place. At the end of this process, affinity-matured plasma cells have developed that continuously secrete antibodies (23). TI B cell responses may be triggered in two ways: TI-2 antigens like polysaccharides efficiently crosslink B cell receptors and initiate a strong and long-lasting antigen-specific primary response (24). In contrast, TI-1 antigens like lipopolysaccharide (LPS) and bacterial DNA (CpG) activate B cells polyclonally, i.e., independent of the B cell receptor, via TLR triggering (25–27). Predominantly B-1- and marginal zone (MZ) B cells can rapidly respond to TI-1 antigens (28).

The main reservoir of B-1 cells are the pleural and peritoneal cavities, but a small proportion can be found in all lymphoid organs. Not only are B-1 cells prone to TI responses, but they are also the main producers of natural antibodies (NAbs), defined as antibodies that circulate in normal individuals in the absence of exogenous antigenic stimulation (29). NAbs are considered polyreactive, usually lack somatic hypermutation and are said to use a restricted set of B cell receptor genes (30–32). NAbs are hence at the interface between innate and adaptive immune responses, and can bridge the time gap until the TD response has matured. In terms of their antigen specificity, B-1 cells are selected for a certain strength of self-antigen binding. Remarkably, ~30% of B-1 cell-derived IgM binds to OSEs. B-1 cells are able to switch to all IgG subclasses *in vitro*, whereas *in vivo*, they are producers of NAbs, mainly of the IgM, IgG3, and IgA isotype [reviewed extensively in (32–34)].

MZ B cells are located close to the marginal sinus in the spleen, where they have direct access to blood-borne antigens (35). Although they have the capacity to generate TD and TI responses, their main function is the TI response against blood-borne pathogens (36, 37). Very early in the course of an infection, they differentiate to IgM- or IgG-secreting cells (38). Both TD as well as TI processes take place during sepsis, and all B cell populations become activated (8, 14). We have therefore tested sepsis-induced IgM- and IgG-binding to a broad panel of bacterial as well as autoantigens. OSE were identified as the dominant target of the B cell response.

## Materials and Methods

### Animals and Ethics Statement

All experiments were performed on 8–12 weeks old female C57BL/6 wild type (WT) mice. All animals were housed in a conventional, temperature-controlled animal facility with a 12-h light and dark cycle, and provided with food and water *ad-libitum*. All experiments were approved by the animal ethics committee of the local animal protection authority (LALLF, State Office for Agriculture, Food Safety and Fisheries Mecklenburg-Western Pomerania; numbers LALLF M-V/TSD/7221.3-1.1-052/07 and LALLF M-V/TSD/7221.3-1.2-013/09). All efforts were made to minimize the suffering of mice.

### CASP Surgery

CASP surgery was performed as described before (39, 40). Briefly, mice were anesthetized with Ketamin (Ketanest, Parke-Davis GmbH, Berlin) and xylazin (Rompun, Bayer Health Care, Leverkusen) intraperitoneally (100 mg/10 mg per kg bodyweight, respectively) and a 18G stent (Ohmeda AB, Helsingborg, Sweden) was implanted into their colon ascendens. Mice were monitored every 4 h until recovery.

### Hybridoma Generation

Splenocytes from mice 10 or 14 days following CASP or sham surgeries were prepared and fused with X63 AG8.653 myeloma cells using polyethylene glycol (Sigma-Aldrich), following an extensive protocol for fusion and selection as described elsewhere (http://www.umass.edu/vetimm/docs/Wagner_Hybridoma.pdf). Briefly, 10 million X63-cells were fused with 1–3 × 10^7^ splenocytes, and the fusion products were plated in several dilutions into 96-well cell culture plates. Ten days later, plates were screened under a light microscope for hybridoma growth. Only hybridomas from plates where <50% of the wells showed cell growth were taken into account, since this improves the probability of monoclonality to over 85% (41). In addition, wells were observed under a light microscope to select for single clone growth based on morphology.

### Immunohistochemical Autoantibody Screening on HEp-2-Cells

Screening of autoantibodies was performed as described before (13). Briefly, serum samples, diluted 1:100 in PBS containing 20% FCS or undiluted hybridoma supernatants were incubated on HEp-2-ANA slides (INOVA Diagnostics, San Diego, CA, USA) overnight at 4°C. Slides were washed with PBS, and bound antibodies were detected either with polyclonal goat anti-mouse IgG or IgM conjugated to FITC (10 μg/mL, Southern Biotech, Atlanta, GA, USA). Slides were then equally exposed, and pictures were taken with a Zeiss Axio imager A.1 fluorescence microscope (Zeiss, Göttingen, Germany) equipped with Spotadvanced software (Diagnostic Instruments, Sterling Heights, MI, USA) using the same settings for all images.

### ELISA

Antigens were prepared for ELISA as follows: (i) Bacterial antigens: Bacteria (*E. coli, E. faecalis, P. mirabilis, S. aureus 8325-4* Δ*spa*) were grown overnight in tryptic soy broth (TSB) medium as described previously (41). Cells were washed twice in cold PBS (3,340 *g* for 15 min at 4°C) and diluted to an optical density at 595 nm (OD_595_) of 1.0 in PBS before being inactivated by irradiation with UV-light for 10 min; (ii) Self-antigens: Histone H2A (calf thymus, 2 μg/mL) and dsDNA (calf thymus, 10 μg/mL), both obtained from Sigma-Aldrich, were diluted in PBS, whereas murine IgG-Fc-fragments (Dianova, 1 μg/mL) were diluted in coating buffer (pH 9.6; Candor Bioscience GmbH, Wangen, Germany); (iii) LPS (*E. coli* O55:B5, Sigma Aldrich, 10 μg/mL) and sepsis unrelated antigens, including TNP (14)-bovine serum albumin (BSA, Biosearch Technologies, 10 μg/mL) and ovalbumin (OVA, Sigma-Aldrich, 10 μg/mL) were diluted in coating buffer to the indicated concentrations; (iv) OSEs: phosphocholine-conjugated BSA (PC-BSA) was obtained from Biosearch Technologies Inc. Malondialdehyde-acetaldehyde-modified BSA (MAA-BSA) was prepared as described elsewhere (42). Human native LDL was isolated, and Cu^2+^-oxidized LDL (CuOx-LDL) and Malondialdehyde-modified LDL (MDA-LDL) were prepared as described previously (42).

96-well flat-bottom microplates (Nunc MaxiSorp™) were coated with the respective antigens overnight at 4°C (bacterial antigens, H2A: 100 μL per well), LPS, dsDNA, murine IgG-Fc-fragments and sepsis unrelated antigens: 50 μl per well). The plates were then washed three times with PBS containing 0.05% Tween20, and blocked with PBS containing 10% fetal calf serum (150 μL/°) for 1 h at room temperature (RT). Murine sera (dilution range 1:100–1:2,500) or hybridoma supernatants were added to the wells and incubated for 90 min at RT. After three washing steps, bound antibodies were detected using F(ab)_2_-fragments of goat anti-mouse IgG or IgM conjugated to POD (Dianova) as previously described (43). Chemiluminescence was measured using a Tecan Sunrise photometer (Tecan Group Ltd., Maennedorf, Switzerland), and the data were expressed as relative light units (RLU) per 100 ms.

### RNA Isolation From Hybridoma Cells and Complementary DNA Generation

Total RNA was isolated from about 1 × 10^5^ hybridoma cells using the RNeasy Mini Kit (QIAGEN GmbH, Hilden, Germany) according to manufacturer's instructions. The isolation steps included a DNase-digestion step to eliminate genomic DNA. The quantity of RNA was determined by using the DS-11 Series Spectrophotometer (DeNovix Inc., DE, USA). One microgram of RNA was transcribed to cDNA using oligo DT Primers of the RevertAid^TM^ First Strand cDNA Synthesis Kit (Fermentas) as per the manufacturer's instructions. For the amplification of V_H_-domain sequences of the immunoglobulins, cDNA was amplified by multiplex PCR using msVHE as universal forward primer and a mixture of the Ig-specific reverse primers (Table 1). The reaction was performed in a total volume of 25 μL using 2 μL of cDNA, 0.2 μL of GoTaq-polymerase (Promega), 2.5 μL of dNTP (1 mM, Roche Diagnostics), 5 μL MgCl_2_ (25 mM, Promega). Primers were used at a final concentration of 200 nM each, and the PCR was performed for 35 cycles at 95°C 30 s, 65°C 30 s, 72°C 45 s and a final elongation step at 72°C for 10 min. PCR products were analyzed on 1.5% agarose gels, and the DNA bands of around 400 bp size were cut under UV light for extraction of DNA using the Nucleo-Spin® Extract II kit (Machery-Nagel).

**Table 1:** Primers used for V_H_-gene sequence amplification.

**Primer designation**	**Primer sequence**	**References**
**Forward Primer:**		
msVHE	5′-GGGAATTCGAGGTGCAGCTGCAGGAGTCTGG-3′	(44)
**Ig-Subclass-Specific Reverse Primers:**		
IgG1	5′-GATCCAGGGGCCAGTGGATAG-3′	
IgG2b	5′-CACCCAGGGGCCAGTGGATAG-3′	
IgG2c inner	5′-GCTCAGGGAAATAACCCTTGAC-3′	(45)
IgG3 inner	5′-GCTCAGGGAAGTAGCCTTTGAC-3′	(46)
IgM	5′-GGCTCTCGCAGGAGACGAGG-3′	

### Cloning and Transformation of Target DNA

The extracted DNA-fragments were cloned into the pCR® 2.1-TOPO® TA vector using the TOPO® TA Cloning® Kit (Life Technologies, Thermo Fisher Scientific, Carlsbad, CA, USA). All steps were done according to manufacturer's instructions. Products were transfected into chemically competent *E. coli* by the heat-shock method. Transformed *E. coli* were cultivated on LB agar plates containing 50 μg/mL of each IPTG, x-Gal and Ampicillin, and incubated at 37°C for 12 h. At least three single white colonies were picked and seeded further into single wells of a 96-well plate containing LB agar with 50 μg/mL Ampicillin.

### V_H_-N-D_H_-N-J_H_ Fragment Sequencing and Analysis

Plasmid isolation and sequencing of the single colonies was performed by GATC Biotech AG, Konstanz, Germany, using the vector-specific M13 forward primer. The resulting sequences were further verified using IMGT/V-Quest database, (https://www.imgt.org/IMGT_vquest/vquest?livret=0&Option=mouseIg).

### Statistical Analysis

Statistical analyses were performed using GraphPad Prism 6 (GraphPad software, San Diego, CA, USA). Data were assessed for significant differences using One-Way ANOVA with Bonferroni *post test* for selected pairs, or using the unpaired Student *t*-test, whenever appropriate. *P* < 0.05 were considered to be significant.

## Results

### A Strong Induction of Total Serum IgG During Sepsis Was Not Due to an Antibacterial IgG Response

**Figure 1:**
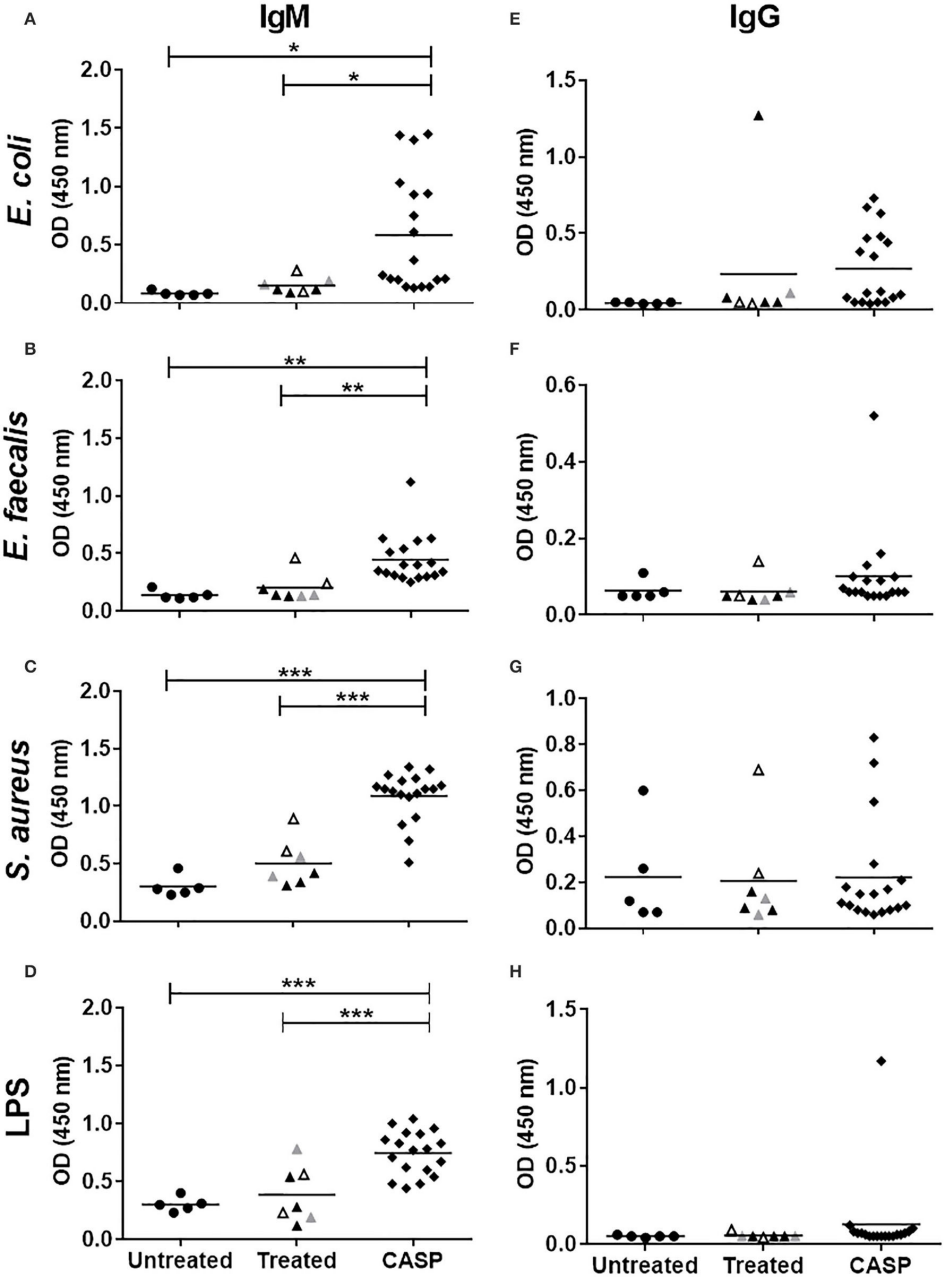
Binding of sera IgM/IgG to bacteria and LPS. The binding of murine serum antibodies, IgM (A-D) or IgG (E-H), to indicated bacteria; *E. coli* **(A,C)**, *E. faecalis* **(B,F)**, *S. aureus* **(C,G)**, and LPS **(D,H)** was tested by ELISA. Fourteen days after sepsis induction, blood was collected, and sera were diluted 1:100 in blocking buffer. Sera from non-septic animals were used as controls. Control mice were untreated (•), whereas the treated mice were anesthetized only (▴), or additionally received a laparotomy (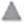), or sham surgery (▵). Mice that underwent CASP surgery are indicated by a black diamond (♦). Mean values of OD 450 nm are shown. Each symbol represents one animal (*N* = 5–18). Statistical analysis was done by One-way ANOVA with the Bonferroni *post test* for selected pairs. *p < 0.05, **p < 0.01, ***p < 0.001.

We and others have shown that sepsis induced a marked increase in serum IgM levels followed by an even stronger IgG response (11, 13). As we have previously shown, most ASCs reside in the spleen, and the class switch to IgG results from both TI and TD processes (14). Since the antigen-specific T cell response is still fully functional at the onset of sepsis (47), we assumed that the class switched IgG response was mainly directed at the invasive bacteria. To test this hypothesis, we induced sepsis in WT mice using the CASP sepsis model. First, systemically disseminated bacteria were identified by plating sera of septic mice onto agar plates followed by microbiological identification of growing colonies. *Enterococcus faecalis (E. faecalis)* and *Escherichia coli (E. coli)*, two microbial species of the intestinal flora, were regularly found in the blood of septic mice. Antibody binding to these bacteria as well as to *Staphylococcus aureus (S. aureus)*, which is often observed in the murine intestine, was then measured by ELISA. UV-inactivated washed bacterial cells served as antigens. To avoid non-specific IgG binding to *S. aureus*, the protein A-deficient mutant strain 8425-Δ*spa* was used. Additionally, LPS of *E. coli* was included. As shown in Figure 1, sepsis induced a significant increase in IgM-binding to whole bacteria as well as LPS. In contrast, there was no significant anti-bacterial or anti-LPS IgG response in the majority of animals. The absence of an anti-bacterial IgG response in sepsis was in striking contrast to immunization with inactivated bacteria (without adjuvant), where ímmunized animals did not develop disease symptoms, but elaborated high specific antibacterial IgG titres (Supplementary Figure 1). Thus, the very strong serum IgG increase in sepsis was not due to an anti-IgG response to the bacteria tested, namely *S. aureus, E. coli* and *E. faecalis*.

### Sepsis-Induced Serum Antibodies Are Directed Against Self-Antigens

**Figure 2:**
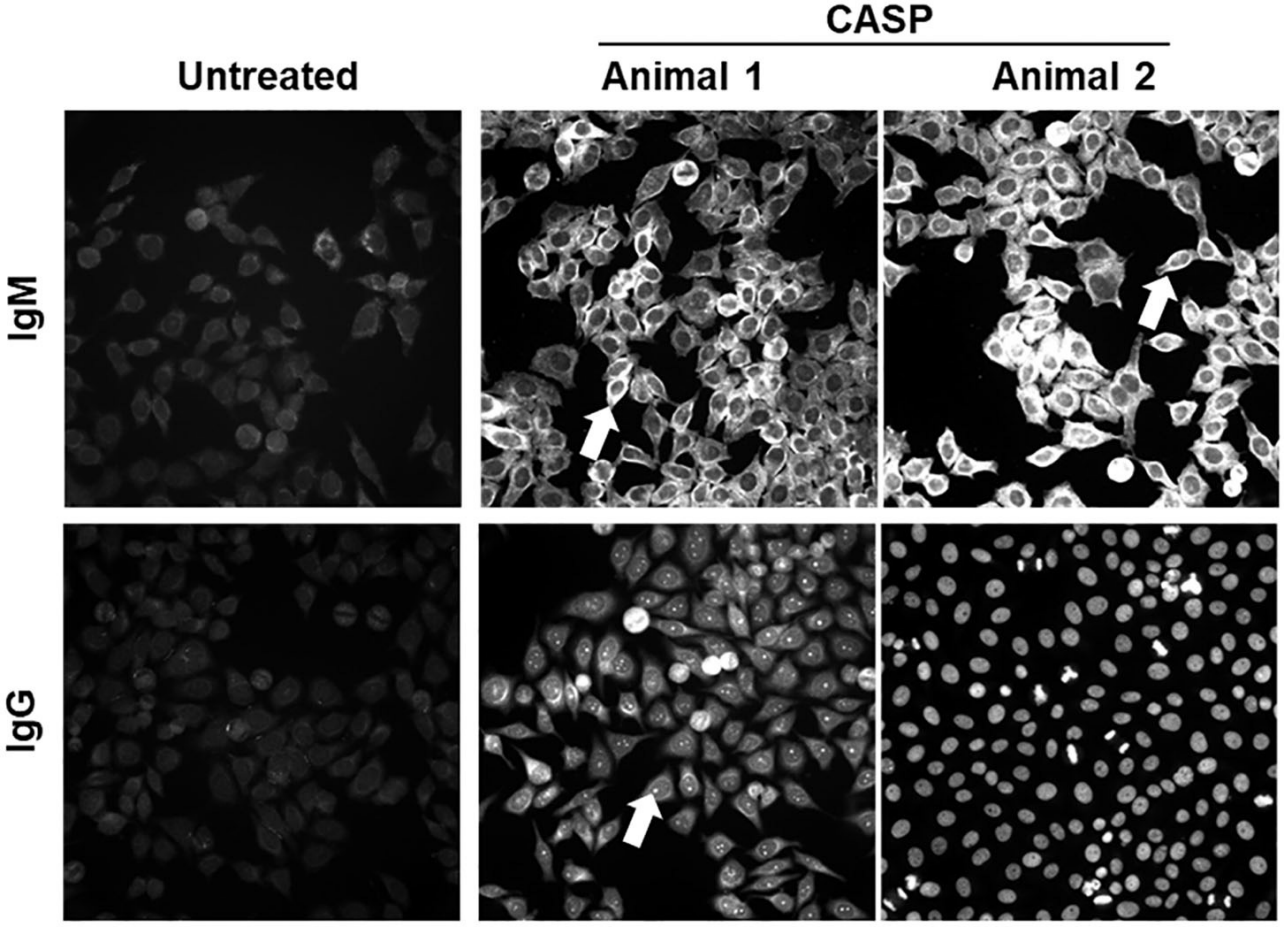
Serum antibodies directed against self-antigens 14 days after sepsis induction. Sepsis was induced in C57BL/6 mice by 18G CASP. Control mice remained untreated. Fourteen days later blood was taken, and sera diluted 1:100 in 20% FCS/PBS were incubated on HEp-2-ANA slides. Bound antibodies were detected by FITC-labeled anti-mouse IgM or IgG antibodies. Out of the tested serum antibodies, 12/13 IgM and 12/13 IgG antibodies were autoreactive. Shown are representative pictures of septic and control mice. *N* = 8 per group. Serum IgM and IgG binding patterns varied greatly. In most cases more than one cellular structure is stained, albeit at variable intensities.

In sepsis, the strong general IgG response with only a modest reaction to bacterial antigens could be caused by polyclonal activation of B-cells due to the release of large amounts of PAMPs and DAMPs. We reasoned that autoreactive Ig should be increased in this case, because the normal B-cell pool contains numerous self-specificities (48–50). Moreover, the PAMPs and DAMPs could also act as adjuvants in a TD immune response to self-antigens that are released from damaged cells and tissues. Therefore, we tested the sera of septic and control mice for Ig-binding to eukaryotic cellular structures using HEp-2 cells as antigens and FITC-labeled anti-mouse-IgM or IgG antibodies for detection of binding. As shown in Figure 2, the sera of septic mice (data from two representative septic animals are depicted) showed an increase in autoreactive IgM and IgG response, as compared to untreated animals (left panel), 14 days after sepsis induction. The fluorescence patterns observed were mostly diverse, where often more than one cellular structure was stained (Figure 2, arrows). The binding patterns differed between individual septic mice from the same cage. Hence, sepsis, which was probably caused by very similar gut microbiota, induced autoantibodies of different specificities. Remarkably, a similar autoreactive response was observed in the sera of splenectomized animals upon sepsis induction (Supplementary Figure 2).

### Sepsis Increased Serum Antibodies Directed Against Sepsis-Unrelated Antigens

An immune response which is largely polyclonal rather than antigen-driven would be expected to show increased antibody binding to antigens unrelated to sepsis. Hence, besides anti-bacterial and self-binding antibodies, we screened for the presence of antibodies binding to such sepsis-unrelated antigens. At first, we investigated the binding of serum antibodies to two antigens the animals had never been exposed to, namely the classical TD protein antigen ovalbumin (OVA) and the hapten 2,4,6-trinitrophenyl (TNP) conjugated to bovine serum albumin (TNP-(14)-BSA). Regarding OVA, only IgM binding increased significantly during sepsis (data not shown), whereas IgM- as well as IgG-binding to TNP-(14)-BSA were both very strongly enhanced (Supplementary Figure 3). Together, these results clearly support the concept of a polyclonal B cell response, which may be dominated by NAb specificities.

### Sepsis Induced High Titer IgM and IgG Antibodies Directed Against Oxidation-Specific Epitopes (OSEs)

**Figure 3:**
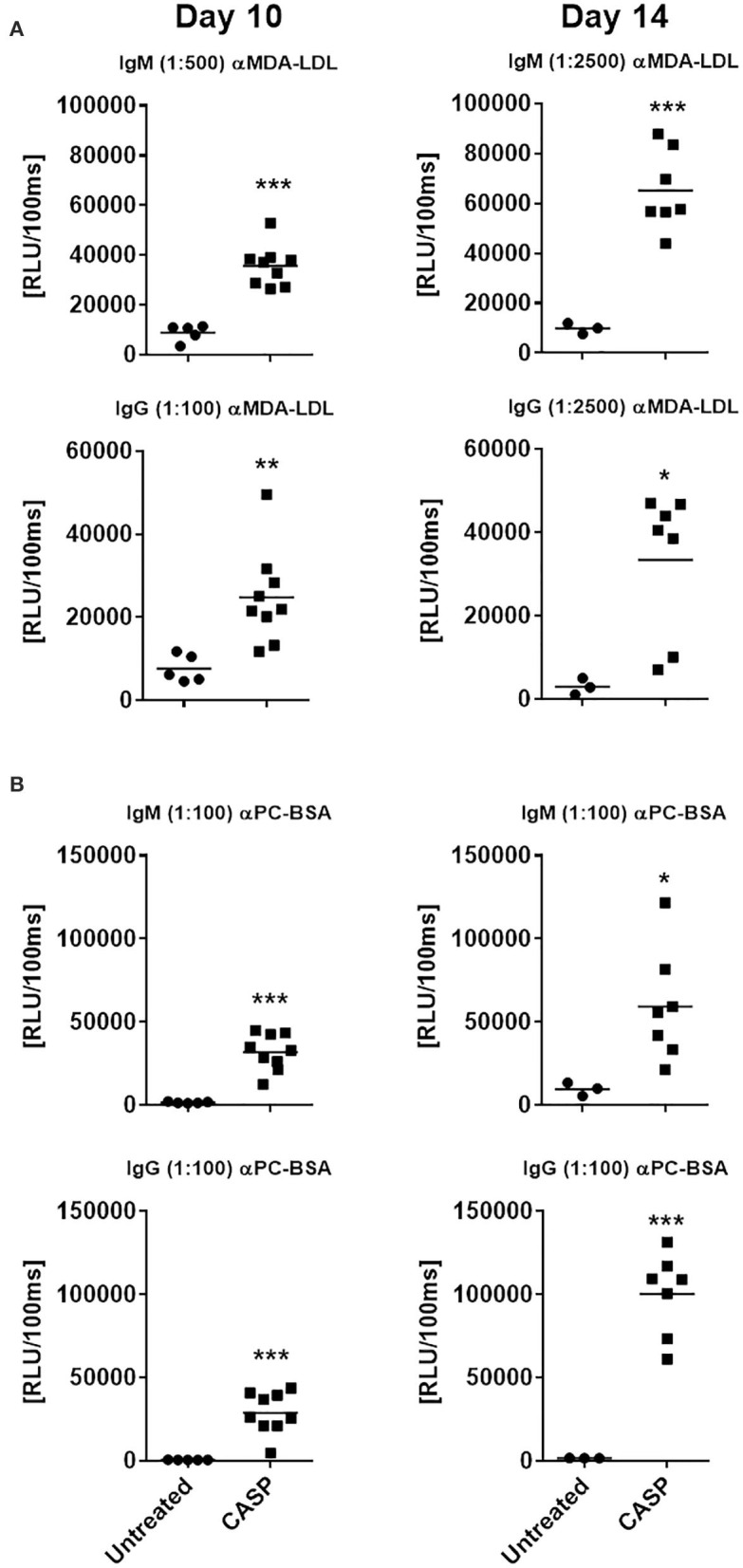
Serum IgM and IgG binding to model oxidation-specific epitopes. C57BL/6 mice were subjected to CASP surgery, while control animals remained untreated. Ten and 14 days after sepsis induction, blood was drawn to determine serum IgM and IgG binding to malondialdehyde-modified LDL [MDA-LDL; **(A)**] and phosphocholine-conjugated BSA [PC-BSA; **(B)**], using chemiluminescent ELISA. Serum dilutions used are indicated in brackets. Statistical analysis was performed using the unpaired *t*-test. *N* = 3–9 per group. *p < 0.05, **p < 0.01, ***p < 0.001.

Since sepsis induced a robust autoreactive-antibody response, we questioned whether these antibodies would be able to bind to OSEs, which are present at high density on apoptotic cells, whose numbers are known to increase during sepsis. OSEs generated during sepsis act as an endogenous DAMPs, and initiate an innate immune response. It has been shown that a large proportion of NAbs, especially those produced by B-1 cells, bind to OSEs (20, 42). Therefore, we tested sera of septic and untreated animals at day 10 and 14 for OSE-specific antibodies by chemiluminescent ELISA. Sepsis elicited a very strong IgM and IgG response to known OSEs in all tested animals (Figure 3 and Supplementary Figure 4), while there were very low levels of OSE-specific antibodies or none at all in untreated animals. Hence, at day 14 post sepsis, the IgM response to both modified LDL and BSA was significantly higher in septic mice than in the control group, where specific IgM was low or even under the limit of detection (Figure 3). On the same day, the IgG/IgM response to MAA-BSA was 4-fold higher than in the untreated group. The IgM response to CuOx-LDL showed a trend toward increased levels, while IgG levels were significantly higher compared to untreated animals (Supplementary Figure 4). Since under other conditions, such as hypercholesterolemia, IgM antibodies dominate the immune response to OSEs (51), the pronounced specific class-switched response in sepsis is remarkable.

### Studies at the Single Cell Level Revealed a High Proportion of Autoreactive IgM Antibodies

To study the sepsis-induced antibodies at a single-cell level, monoclonal antibodies (mAbs) were generated. Since we discovered the spleen as the main source of antibody secreting cells (ASCs) in sepsis (14), splenocytes from eight septic mice were obtained on day 10 after sepsis induction and fused. Three quarters (74.3%) of the hybridomas from septic mice produced IgM (Table 2). However, sepsis induced a much stronger increase in serum IgG than IgM, which peaked at day 14 (14). Assuming that the class switch to IgG may not have been completed on day 10 after sepsis induction, additional fusions were performed using splenocytes from six septic mice on day 14. However, choosing this later time point did not change the proportion of IgM- to IgG-producing cells, where 82% of the resulting hybridomas secreted IgM (Table 2). Several attempts to produce hybridomas from non-septic control mice failed, presumably due to the lack of activated B cells and plasmablasts in the spleen, which is a limitation of this study (data not shown). Determination of the mAb specificities confirmed and extended the observations made with serum antibodies, namely that sepsis induces autoreactive antibodies, predominantly IgM.

**Table 2:** HEp-2-positive IgM- or IgG-secreting hybridomas.

	**HEp-2-positive hybridoma supernatants**			
	**IgM**		**IgG**	
	***N*/total**	**%**	***N*/total**	**%**
CASP d10	91/225	**40**	3/78	**4**
CASP d14	21/68	**31**	0/15	**0**

*The table shows the numbers of IgM or IgG hybridoma supernatants binding to cellular structures of HEp-2 cells, respective to the total numbers of tested hybridomas. Splenocytes were obtained on day 10 (eight animals, eight fusions) or 14 (six animals, seven fusions) after sepsis induction*.

**Figure 4:**
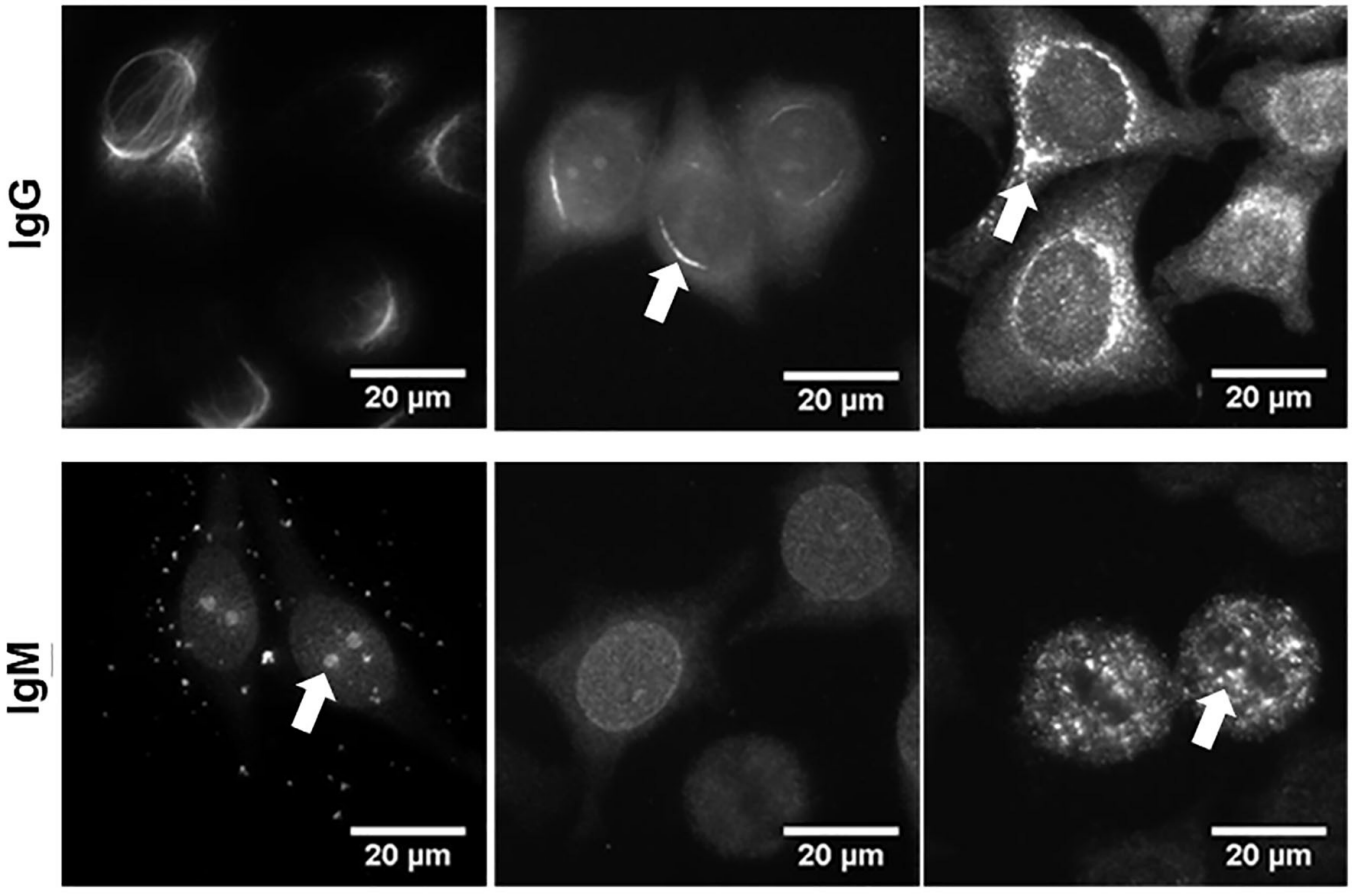
Monoclonal antibodies derived from splenic B cells of septic mice bind several cellular self-antigens. Ten or 14 days after CASP induction, splenocytes were isolated and fused with myeloma cells to obtain hybridomas. Supernatants of monoclonal IgM- or IgG-producing hybridomas were incubated on HEp-2-ANA slides to detect auto-reactivity. Bound IgG or IgM was detected by FITC-labeled anti-mouse IgG or IgM antibodies. Three examples, respectively, of autoreactive monoclonal IgM- and IgG-antibodies are shown. The binding patterns of the various antibodies showed obvious differences, where in most cases more than one cellular structure was stained, albeit at varying intensities (indicated by arrows). For quantified data, regarding the numbers/percent of IgM or IgG hybridoma supernatants from septic mice directed against self-antigens, please refer to Table 2.

The total of 386 hybridoma supernatants derived from the 14 above mentioned septic mice and containing either IgM or IgG mAbs were screened for autoreactivity. A large proportion of IgM mAbs (31% to 40%) reacted with specific structures of the HEp-2 cells (Table 2). HEp-2-binding IgM mAbs were found in every animal, regardless of whether the spleen cells were obtained 10 or 14 days after sepsis. In contrast, only three out of 93 IgG mAbs showed detectable HEp-2-binding, when using undiluted hybridoma culture supernatants. These auto-reactive IgG hybridomas were obtained from three mice. Figure 4 illustrates the variability of the binding patterns of the mAbs derived from splenic B cells of septic mice 10 or 14 days after CASP induction, which bind several cellular self-antigens. FITC-labeled anti-mouse IgG or IgM antibodies were used to determine the binding of those mAbs and clearly showed that, similar to our previous experiment at the serum level, the binding patterns of the individual antibodies were highly variable.

### Most IgM Antibodies Are Polyreactive

Since we observed high titres of autoantibodies in sera of septic mice at day 10 or 14 (Figure 2), we wondered whether this also applies to the mAbs present in the hybridoma supernatants of six septic mice (generated from splenocytes at day 10 of sepsis).

We tested the hybridoma supernatants for mAb-binding to a broad panel of antigens, including bacterial and self-antigens, as well as two antigens that the animals had never been exposed to, namely TNP and OVA. Binding was observed with 27 out of 120 IgM-supernatants (22.5%) (Table 3) but with none of the 40 tested IgG supernatants (data not shown). The majority of the binding IgM mAbs (85%) were polyreactive, recognizing at least two antigens in the test panel used.

**Table 3:** Antigen-binding patterns of positively-tested IgM hybridomas.

	**Positive clones**	**Bacterial antigens**				**Foreign antigens**		**Self antigens**			
		***E. coli***	***P. mirabilis***	***S. aureus***	**LPS (*E. coli)***	**TNP-BSA**	**OVA**	**ds DNA**	**histone HIIA**	**IgG-Fc**	**HEp-2-Test**
1	F4_03		+							+	/
2	F4_04					+					+
3	F4_05			+		+		+	+	+	+
4	F4_09		+							+	+
5	F4_16	+	+			+				+	+
6	F4_23	+	+	+	+	+	+	+	+	+	+
7	F4_38			+				+	+		+
8	F4_46									+	+
9	F4_55					+					/
10	F5_10					+					+
11	F5_11	+		+						+	+
12	F5_17	+	+							+	
13	F6_20									+	
14	F7_07									+	+
15	F7_30					+					+
16	F7_32					+					+
17	F7_33					+					+
18	F7_36			+							+
19	F7_39					+					
20	F8_14					+				+	+
21	F9_09		+							+	+
22	F9_10		+			+				+	+
23	F9_12		+						+	+	/
24	F9_24			+		+				+	+
25	F9_29					+					+
26	F9_33					+					
27	F9_38			+		+		+		+	+

*Overview of the antigen binding patterns of 27 binding (out of a total of 120 tested) IgM hybridoma-supernatants. The hybridomas were generated from splenocytes of septic mice at day 10 and tested for binding to a panel of bacterial, sepsis-unrelated (foreign) as well as self-antigens by ELISA. Binding to HEp-2 cells was tested by fluorescence microscopy. (+, binding; /, not tested). The corresponding OD (450 nm) values are shown in Supplementary Table 1*.

### In Septic Mice, a Large Proportion of mAbs, Both IgM and IgG, Binds to Oxidation Specific Epitopes

Eighteen IgM mAbs that showed reactivity to the antigen panel in Table 3, as well as all available IgG mAbs (*N* = 20) of so far unknown specificity, were tested for binding to OSEs. Thirteen (72%) IgM and 5 (25%) IgG mAbs showed binding to one or several OSEs in ELISA (Table 4). Examples of IgG mAb titration curves are shown in the Supplementary Figure 5. The five OSE-specific IgG hybridomas were derived from four different mice. Remarkably, with the exception of IgG3, the OSE-specific mAbs encompassed all IgG subclasses. These findings underline that OSEs are prominent targets of the humoral immune response in sepsis.

**Table 4:** Binding pattern of CASP d10 monoclonal IgG and IgM to oxidation-specific epitopes.

**Clone**	**Ig-class**	**Binding specificity**			
		**Oxidation-specific epitopes (OSE)**			**Human native LDL**
		**MAA-BSA**	**MDA-/CuOx-LDL**	**PC-BSA**	
F4_32	IgG1	×	×	ND	-
F6_13	IgG2b	×	×	-	-
F8_07	IgG2c	×	×	×	×
F9_3_H7	IgG1	×	×	ND	-
F9_25	IgG2c	×	×	-	-
F4_38	IgM	×	×	-	-
F5_10	IgM	×	×	×	-
F7_07	IgM	×	×	-	-
F9_24	IgM	×	×	-	-
F9_38	IgM	×	×	×	-
F4_04	IgM	×	×	-	-
F4_16	IgM	×	×	-	-
F4_46	IgM	×	-	-	-
F7_32	IgM	×	×	-	-
F9_09	IgM	×	-	-	-
F9_29	IgM	×	×	-	-
F8_14	IgM	×	×	×	×
F9_10	IgM	×	×	×	×
F7_33	IgM	-	-	-	×
F4_09	IgM	-	-	-	-
F7_36	IgM	-	-	-	-
F7_39	IgM	-	-	-	-
F9_12	IgM	-	-	-	-

*The hybridomas were generated from splenocytes of septic mice at day 10. Twenty IgG hybridoma-supernatants (diluted to 0.25 μg/mL) and 18 IgM hybridoma-supernatants (diluted to 0.125 μg/mL) were tested for binding to model oxidation-specific epitopes. Human native LDL served as control. Binding specificities of five positively-tested IgG, as well as all IgM clones, are shown. ×, binding; -, no binding; MAA-BSA, malondialdehyde-acetaldehyde-modified bovine serum albumin (BSA); MDA/CuOx-LDL, malondialdehyde-modifed (MDA)/Cu^2+^-oxidized (CuOx)-LDL; PC-BSA, phosphorylcholine conjugated to BSA; ND, not determined*.

### Most Sepsis-Induced IgG mAbs Carried Few Somatic Mutations

**Figure 5:**
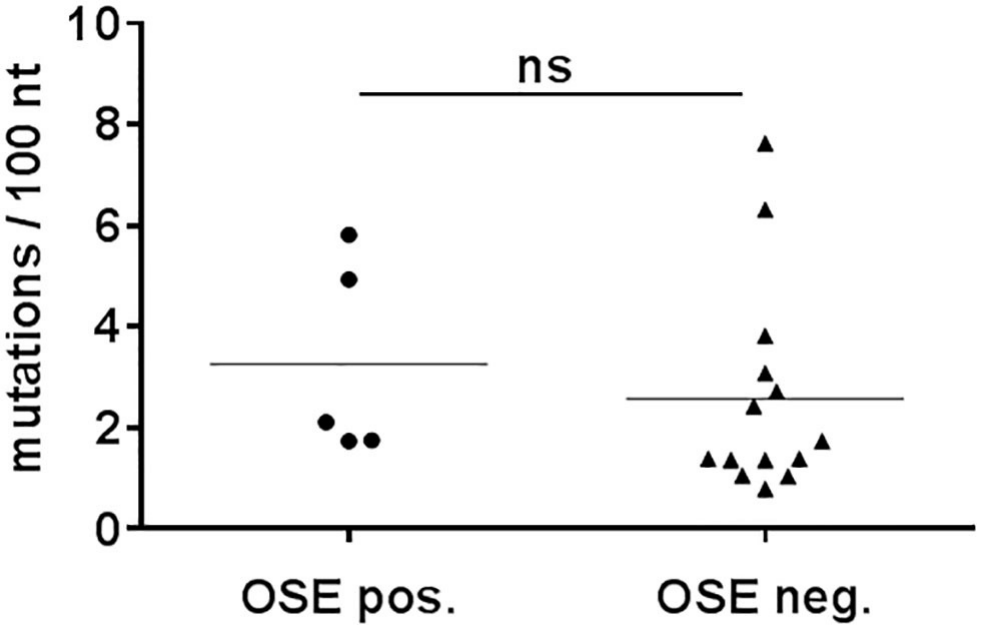
Number of mutations in Vh-genes of monoclonal IgG. CASP surgery was performed on C57BL/6 mice. Hybridomas were generated by fusing splenocytes from 10- days-septic mice. The resultant monoclonal IgG hybridomas were sequenced for Vh-genes. Mutations of monoclonal IgG that showed binding to OSEs (OSE pos.) were compared to IgG that showed no binding (OSE neg.). Statistical analysis was performed using the unpaired *t*-test. *N* = 5–14 per group. nt, nucleotides; ns, not significant.

Many class switched antibody responses are TD and are characterized by somatic hypermutation. Therefore, the IgG mAbs were tested for somatic hypermutation by sequencing the variable domains of the IgG heavy chains. IgG mAbs at day 10 or 14 after sepsis mostly showed no significant differences in mutations (Supplementary Figure 6A). Most sequences were near germline, similar to 12 IgG monoclonal antibodies obtained from an untreated animal, which all had <4 mutations/100 nucleotides (Supplementary Figure 6A). However, four out of the 34 IgG mAbs derived from septic animals had five or more mutations per 100 nucleotides, indicative of somatic hypermutation. As such mAbs were present already at day 10, they were probably derived from memory B cells (52). OSE-specific and –non-specific IgG mAbs showed similar proportions of highly mutated IgG (Figure 5). Thus, most of the IgG antibodies generated during sepsis used near-germline gene sequences. Regarding the CDR3 length, the number of amino acid exchanges per V-region as well as the proportion of non-silent mutations, there were no significant differences between septic and non-septic animals (Supplementary Figures 6, 7).

## Discussion

Our data clearly show that OSEs are the main targets of the strong humoral immune response observed in sepsis. While Ig binding patterns to other self-epitopes were individual, OSE-specific binding was observed in all tested animals.

The sepsis-induced suppression of the adaptive immune system is well-documented (10, 13, 53). Nevertheless, we and others observed a strong increase in serum IgM and IgG concentrations following sepsis (13, 14). Interestingly, the peak of the IgG serum concentration exceeded that of IgM by a factor of four, and an Ig switching to all IgG subclasses was seen. However, at the single cell level, there were three times as many IgM hybridomas as those producing IgG. Although one could argue that fusion was performed too soon, we found essentially the same proportions on day 14 as on day 10, which rules out time as the causal factor. Although the numbers of hybridomas are in general too low to make a definitive conclusion, a plausible explanation for this observation may be found in the half-lives of IgM vs. IgG antibodies. IgM has a markedly shorter serum half-life than IgG, and the half-life of polyreactive IgM is further reduced by Ag-binding, leading to a relative accumulation of IgG (54, 55).

Since sepsis induces dissemination of endogenous bacteria into the bloodstream (11), a specific antibacterial IgG response would have been expected. However, there was very little serum IgG binding to bacteria in post-septic mice. Since we focussed our investigation on culturable bacteria in the sera of septic mice, it is possible that bacterial species other than the ones examined here were the culprits, since ELISA was performed only for *E. coli, S. aureus*, and *E. faecalis*. However, *E. coli* and *E. faecalis* originate from the serum of a septic animal. In fact, the bacterial strains used for testing are definitely present in large numbers in the intestine. When animals were injected *i.p*. with dead bacteria (*E. coli, S. aureus*, and *E. faecalis*) without sepsis, they were perfectly able to elaborate a specific IgG response. Depending on the bacterial species, we could show that 10^7^-10^9^ UV-inactivated bacteria trigger a robust specific IgG response (Supplementary Figure 1). Hence, we assume that sepsis interferes with an antibacterial antibody response. Interestingly enough, we rather observed Ig binding to autoantigens, as well as antigens, to which the animals had never been exposed. The production of natural autoantibodies against a spectrum of autoantigens has been described in sepsis patients (56–58). Using a relatively small panel of autoantigens, Burbelo et al. detected autoantibodies in 46% of severe sepsis patients, suggesting that this is a relatively common phenomenon (58). However, the production of autoantibodies is not confined to sepsis, but appears to be a general phenomenon observed with other types of bacterial, viral, and protozoan infections (18, 59–61), in addition to autoimmune disorders. In their recent study, Sakakibara et al. observed that the immune response to murine γ-herpesvirus 68 is accompanied by autoantibody production from polyreactive B cells in the germinal center, whose self-reactivity is generated through somatic hypermutation (61). More interestingly, several reports suggest a role of polyclonal activation in triggering anti-self responses, and hence leading to autoimmune disorders as a consequence of infections (18, 62). For instance, a direct link between infection and autoimmune encephalitis has been experimentally demonstrated in mice, and has been attributed to the production of autoantibodies (62).

It has been demonstrated that sepsis induces massive apoptosis of B and T lymphocytes, as well as DCs in mice and humans, and the release of danger molecules, known as PAMPs and DAMPs (8, 9, 63–67). We confirmed this in our model by TUNEL staining (terminal deoxynucleotidyl transferase dUTP nick end labeling) of the spleens for apoptotic nuclei (data not shown). The observed autoantigen response in sepsis could be explained by a polyclonal B cell activation through PAMPs and DAMPs, which activate B cells independent of the BCR, leading to the production of the so-called NAbs, which are polyreactive (68). To test whether polyreactive (natural) Abs or monoreactive Ig from a large variety of B cells dominate the humoral immune response after sepsis, an analysis at the single cell level was conducted. It revealed that a large proportion of IgM-producing cells were polyreactive, as one would have expected based on the results obtained with serum.

Our results identify OSEs as the dominant target of the B cell response in murine polymicrobial sepsis: First, all post-septic animals had OSE-binding IgM and IgG, whereas antibody binding patterns to other tested autoantigens differed between individuals, even from the same cage. Second, anti-OSE titres were much higher than those of other tested bacterial or self-antigens. Finally, OSE-specific B cells were very frequent. Thirteen out of 18 tested IgM mAbs bound to OSEs even at low concentrations. Since 19% (23/120) of the IgM mAbs were polyreactive, at least 14% of IgM-producing B cells were OSE-specific. This frequency is somewhat similar to that of IgG mAbs, of which 25% were OSE-reactive.

Several points argue for a B-1 cell-driven immune response in the case of IgM. B-1 cells reside in the peritoneum and upon activation migrate to the spleen as well as other lymphatic organs, where they differentiate into ASCs (69, 70). These are the main producers of NAbs, which are polyreactive and utilize germline-near sequences (68). Chou et al. have shown that 20–30% of B-1 cell-derived IgM targets model-OSEs (42). This reflects the frequencies we find in monoclonal IgM and IgG. Moreover, Chang et al. have demonstrated that immunization of mice with apoptotic cells, which are abundant in sepsis, induces OSE-binding IgM (70).

In contrast, it is rather unlikely that the IgG response to OSEs is also dominated by B-1 cells. In B-1 cells, only a switch to IgG3 has been shown *in vivo* (31, 71). However, none of the five OSE-specific IgG mAbs were IgG3, whereas all other IgG subclasses were represented. Hence, a TD response of follicular B cells appears more likely. Chang et al. were able to induce OSE-binding IgG in mice by immunizing with model-OSE and apoptotic cells simultaneously (70). Moreover, Grasset et al. showed that some follicular B cells can bind fluorescently-labeled oxidized LDL. Repeated immunization with apoptotic cells induced OSE-binding IgG (72).

We suggest the following model: In sepsis, numerous immune cells undergo apoptosis. PAMPs and DAMPs polyclonally activate peritoneal B-1 cells to migrate to the spleen and differentiate into ASCs. These produce NAbs, explaining the increase in serum IgM targeting OSEs as well as other autoantigens. NAbs, including OSE-targeting IgM antibodies, may act as scavengers, promoting the clearance of apoptotic cells and debris, which would be relevant in sepsis (22, 42). In this study, however, we observed that OSE-specific antibodies had switched to IgG as well, presumably in a TD response. It may be speculated that the dominant response to OSEs restricts a specific antibody response to the sepsis-causing bacteria, which the organism would mount in the absence of apoptosis. Besides this, massive apoptosis has been shown to be tolerogenic, which explains the well-known fact that sepsis does not induce strong protective immune memory (11). The pathophysiological function of OSEs and OSE-specific IgM and IgG in sepsis should now be investigated, to determine whether OSE-specific antibodies could have therapeutic potential.

## Data Availability Statement

All datasets generated for this study are included in the article/Supplementary Material.

## Ethics Statement

The animal study was reviewed and approved by The Animal Ethics Committee of the local animal protection authority (LALLF, State Office for Agriculture, Food Safety, and Fisheries Mecklenburg-Western Pomerania).

## Author Contributions

ON, CP, CB, and BB: conceptualization and project design. ON, CP, JL, SQ, and AL: methodology and performance of experiments. ON and CP: data evaluation. ON, CP, DR, C-DH, MD, and BB: interpretation of data. ON, DR, MD, CJB, and BB: writing—original draft preparation. All authors critically reviewed the manuscript and approved the submitted version.

## Conflict of Interest

The authors declare that the research was conducted in the absence of any commercial or financial relationships that could be construed as a potential conflict of interest.
