# Supplementary material for: Oxidation-Specific Epitopes (OSEs) Dominate the B Cell Response in Murine Polymicrobial Sepsis
Source: Front Immunol. 2020 Jul 31;11:1570. doi: 10.3389/fimmu.2020.01570 (PMC7412885; doi:10.3389/fimmu.2020.01570)
Supplement: Supplementary file 1 [file Data_Sheet_1.docx]

Supplementary Material

# Supplementary Figures

**Supplementary Figure 1. Humoral IgG response 14 days after *i.p*. application of UV-inactivated bacteria without adjuvant.**

C57BL/6-mice were intraperitoneally injected with 1×10^7^ (●). 1×10^8^ (▼) or 1×10^9^ (■) colony forming units (CFU) UV-inactivated bacteria. *E. coli* (A). *E. faecalis* (B) or *S. aureus* JSNZ (C). Blood was collected before (D0) and two weeks after injection (D14). Sera were tested for IgG-binding to the injected bacteria. and the values. together with the median. are presented as arbitrary units (aU). Each symbol represents one animal; N = 3 per group.

**IgM**


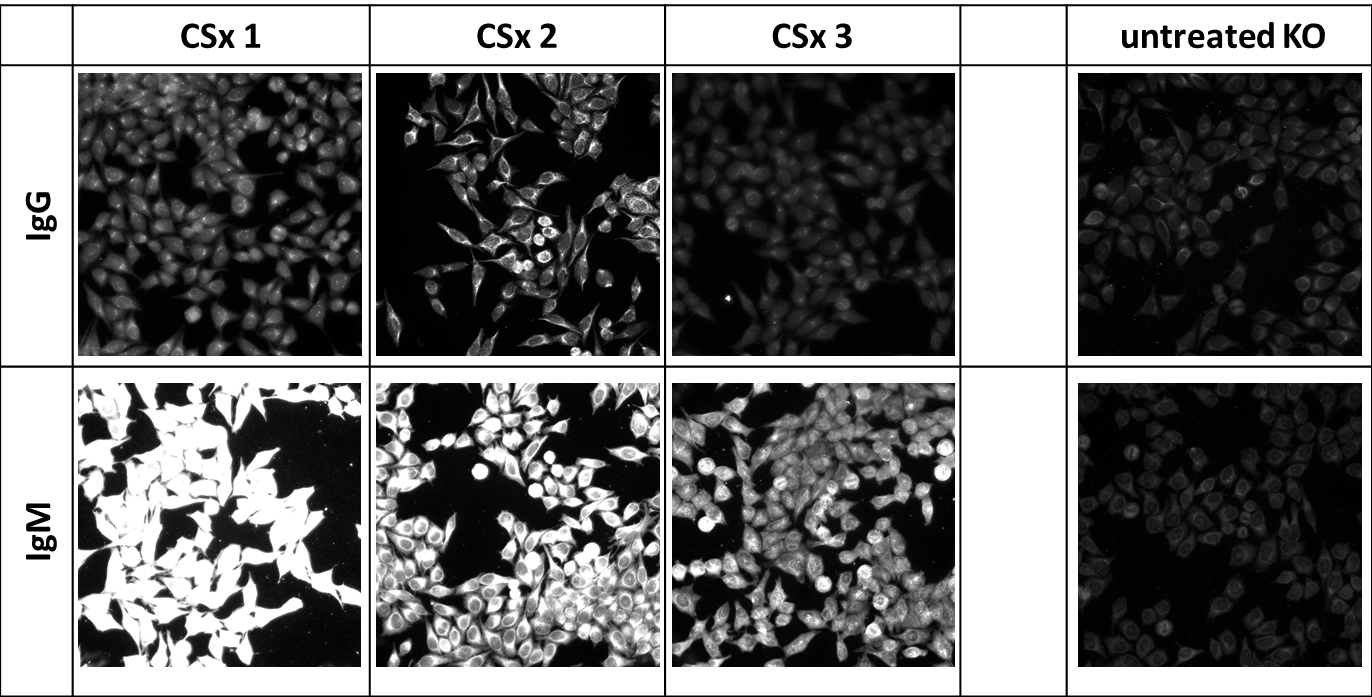

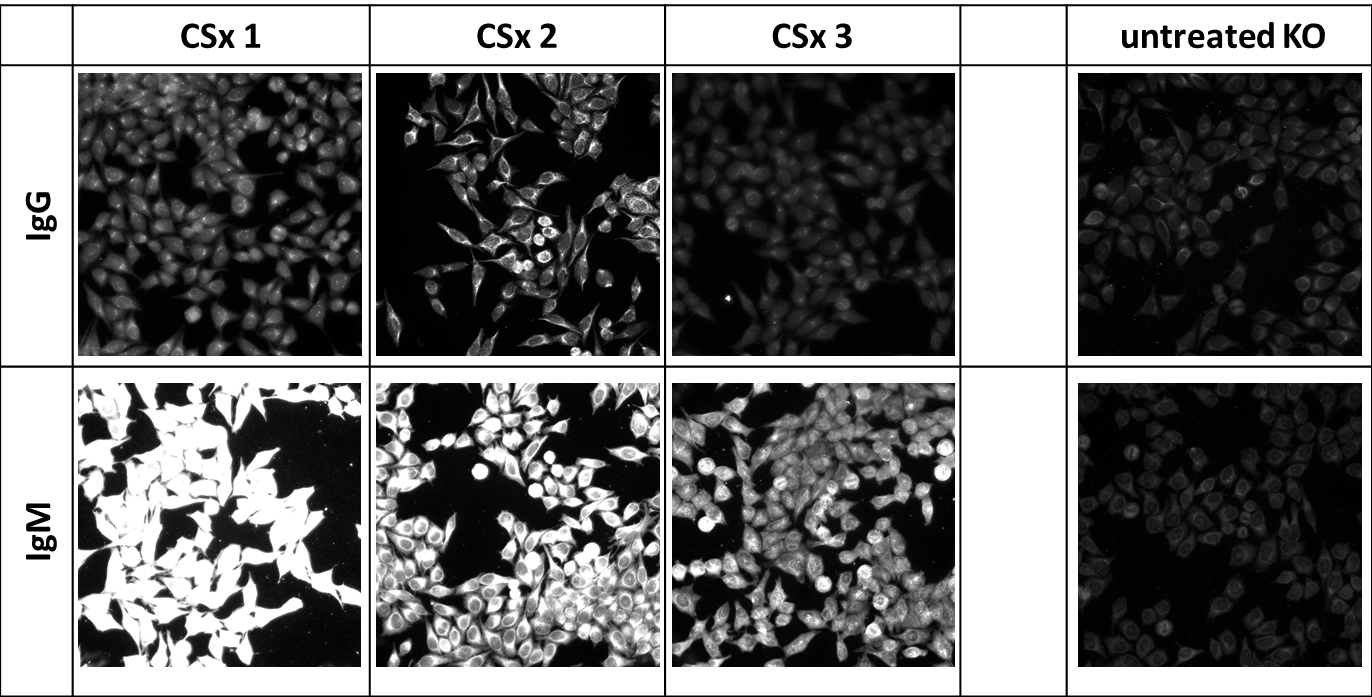


**Animal 1 Animal 2 Animal 3 Untreated**

**IgG**

**CASP**

**Supplementary Figure 2. Autoreactive IgM or IgG in the absence of marginal zone B cells.** Mice were splenectomized and CASP was induced as described in the methods section. Blood was collected at day 14 post-sepsis. and the sera were diluted 1:100 in 20% FCS/PBS and incubated on HEp-2-ANA slides. Bound antibodies were detected by FITC-labeled anti-mouse IgM or IgG antibodies. Sepsis in splenectomized mice showed increased IgM response as compared to untreated animals. IgM and IgG bound to cytoplasmic and nuclear structures (indicated by arrows), respectively, as judged by the observed fluorescence pattern.

**Supplementary Figure 3. Serum Ig binding to sepsis-unrelated antigens 14 days after CASP.**

CASP was performed on C57BL/6 mice as previously described. Untreated animals served as a control. Fourteen days after sepsis induction. blood was taken and the amount of TNP-(14)-BSA-specific serum IgM and -IgG was determined by ELISA. The values are presented in arbitrary units (au). Mice had not been in contact with TNP-(14)-BSA at any time point. Statistical analysis was performed using the unpaired t-test. N = 15-22 per group; *** p <0.001

**Supplementary Figure 4. Serum IgM and IgG binding to malondialdehyde-acetaldehyde–modified BSA (MAA-BSA)**

C57BL/6 mice were subjected to CASP surgery as described in the methods sections. Control animals remained untreated. Fourteen days after sepsis induction blood was withdrawn. and the serum IgM- and IgG-binding to MAA-BSA was determined by ELISA. Serum dilutions are indicated in brackets. Statistical analysis was performed using the unpaired t-test. N = 3-7 per group; **p<0.01. *** p <0.001

**Supplementary Figure 5. Titration curves of monoclonal IgG binding to OSE**

Monoclonal IgG derived from hybridomas generated from splenocytes of post-septic mice (D10) were testetd for binding to OSE by ELISA. Titration curves for two IgG mAb binding to MDA-LDL and nat. LDL (control) are shown.

**Supplementary Figure 6. Sequence analysis of Vh-genes of monoclonal IgG**

CASP was performed in C57BL/6 mice as previously described. Vh-genes of monoclonal IgG hybridomas were obtained by fusing spleen cells either from mice that were left untreated, or from mice 10 (D10) or 14 days (D14) post sepsis, and then sequenced. Statistical analysis was done by One-way ANOVA with the Bonferroni Post Test for selected pairs. Mean values are shown. N = 8-26 per group.

**Supplementary Figure 7. Mutation analysis in Vh-genes of monoclonal IgG**

Sepsis was induced in C57BL/6 mice by CASP surgery. Vh-genes of monoclonal IgG hybridomas obtained by fusing spleen cells either from mice that were left untreated, or from mice 10 or 14 days after sepsis induction, were sequenced. Untreated-IgG (N = 11); CASP D10-IgG (N = 26); CASP D14-IgG (N = 8).

# Supplementary Tables

**Supplementary Table 1. Overview of the antigen binding patterns of 27 out of 120 total tested IgM hybridoma-supernatants.**

Hybridomas were generated from splenocytes of septic mice at day 10 following sepsis induction, and were tested for binding to a panel of bacterial antigens, self-antigens and sepsis-unrelated antigens by ELISA. Binding to HEp-2 cells was tested by fluorescence microscopy. (+, binding; /, not tested). Shown are the optical densities (OD) measured at 450 nm. Background OD values for each antigen are given in brackets. Values in bold with a grey background are those above an arbitrary threshold value set at ≥ 2× background OD.

|  |  | **Bacterial antigens** | | | |  | **Foreign antigens** | |  | **Self antigens** | | |  |  |
| --- | --- | --- | --- | --- | --- | --- | --- | --- | --- | --- | --- | --- | --- | --- |
|  | **pos. clones** | ***E. coli*** | ***P. mirabilis*** | ***S. aureus*** | **LPS  (*E. coli*)** |  | **TNP-BSA** | **OVA** |  | **dsDNA** | **histone HIIA** | **IgG-Fc** |  | **HEp-2-Test** |
|  |  | **(0.08)** | **(0.08)** | **(0.1)** | **(0.05)** |  | **(0.06)** | **(0.05)** |  | **(0.05)** | **(0.05)** | **(0.08)** |  |  |
| **1** | **F4_03** | 0.11 | **0.16** | 0.15 | 0.05 |  | 0.06 | 0.05 |  | 0.05 | 0.05 | **0.17** |  | **/** |
| **2** | **F4_04** | 0.09 | 0.09 | 0.12 | 0.05 |  | **1.21** | 0.06 |  | 0.06 | 0.06 | 0.13 |  | **+** |
| **3** | **F4_05** | 0.11 | 0.12 | **0.22** | 0.05 |  | **0.12** | 0.06 |  | **1.07** | **0.73** | **0.16** |  | **+** |
| **4** | **F4_09** | 0.12 | **0.17** | 0.16 | 0.05 |  | 0.05 | 0.06 |  | 0.06 | 0.06 | **0.18** |  | **+** |
| **5** | **F4_16** | **0.18** | **0.22** | 0.18 | 0.06 |  | **0.2** | 0.05 |  | 0.05 | 0.05 | **0.33** |  | **+** |
| **6** | **F4_23** | **0.85** | **0.95** | **1.06** | **1.07** |  | **1.45** | **0.31** |  | **0.54** | **0.91** | **0.92** |  | **+** |
| **7** | **F4_38** | 0.09 | 0.1 | **0.21** | 0.05 |  | 0.07 | 0.04 |  | **1.37** | **1.43** | 0.1 |  | **+** |
| **8** | **F4_46** | 0.1 | 0.11 | 0.14 | 0.05 |  | 0.07 | 0.04 |  | 0.08 | 0.07 | **0.31** |  | **+** |
| **9** | **F4_55** | 0.09 | 0.1 | 0.12 | 0.05 |  | **0.39** | 0.04 |  | 0.05 | 0.05 | 0.09 |  | **/** |
| **10** | **F5_10** | 0.1 | 0.11 | 0.13 | 0.05 |  | **0.92** | 0.05 |  | 0.05 | 0.05 | 0.14 |  | **+** |
| **11** | **F5_11** | **0.17** | 0.14 | **0.2** | 0.05 |  | 0.06 | 0.04 |  | 0.05 | 0.05 | **0.18** |  | **+** |
| **12** | **F5_17** | **0.2** | **0.21** | 0.18 | 0.05 |  | 0.06 | 0.05 |  | 0.05 | 0.04 | **0.25** |  | - |
| **13** | **F6_20** | 0.11 | 0.13 | 0.13 | 0.04 |  | 0.05 | 0.05 |  | 0.05 | 0.05 | **0.16** |  | - |
| **14** | **F7_07** | 0.13 | 0.14 | 0.13 | 0.05 |  | 0.05 | 0.05 |  | 0.05 | 0.06 | **0.16** |  | **+** |
| **15** | **F7_30** | 0.08 | 0.09 | 0.1 | 0.05 |  | **0.32** | 0.06 |  | 0.07 | 0.06 | 0.08 |  | **+** |
| **16** | **F7_32** | 0.09 | 0.1 | 0.12 | 0.04 |  | **0.26** | 0.05 |  | 0.06 | 0.06 | 0.1 |  | **+** |
| **17** | **F7_33** | 0.09 | 0.1 | 0.12 | 0.05 |  | **0.19** | 0.05 |  | 0.05 | 0.05 | 0.1 |  | **+** |
| **18** | **F7_36** | 0.1 | 0.11 | **0.59** | 0.05 |  | 0.05 | 0.06 |  | 0.05 | 0.05 | 0.1 |  | **+** |
| **19** | **F7_39** | 0.09 | 0.09 | 0.1 | 0.04 |  | **0.31** | 0.06 |  | 0.06 | 0.05 | 0.1 |  | - |
| **20** | **F8_14** | 0.1 | 0.11 | 0.13 | 0.05 |  | **1.36** | 0.06 |  | 0.06 | 0.05 | **0.17** |  | **+** |
| **21** | **F9_09** | 0.13 | **0.16** | 0.16 | **0.48** |  | 0.05 | 0.07 |  | 0.05 | 0.05 | **0.16** |  | **+** |
| **22** | **F9_10** | 0.15 | **0.17** | 0.15 | 0.06 |  | **0.77** | 0.06 |  | 0.06 | 0.06 | **0.18** |  | **+** |
| **23** | **F9_12** | 0.14 | **0.2** | 0.19 | 0.05 |  | 0.06 | 0.06 |  | 0.06 | **0.33** | **0.18** |  | **/** |
| **24** | **F9_24** | 0.13 | 0.15 | **0.2** | 0.05 |  | **0.15** | 0.06 |  | 0.06 | 0.08 | **0.26** |  | **+** |
| **25** | **F9_29** | 0.11 | 0.1 | 0.12 | 0.05 |  | **0.94** | 0.06 |  | 0.06 | 0.05 | 0.11 |  | **+** |
| **26** | **F9_33** | 0.1 | 0.1 | 0.11 | 0.04 |  | **0.19** | 0.06 |  | 0.08 | 0.04 | 0.08 |  | - |
| **27** | **F9_38** | 0.13 | 0.15 | **0.2** | 0.06 |  | **1.4** | 0.05 |  | **0.14** | 0.06 | **0.17** |  | **+** |

**Supplementary Table 2. Mutation frequencies in the V-region of Vh of monoclonal IgG. generated 10 days after CASP.**

Comparison of OSE-posive and OSE-negative monoclonal IgG antibodies derived from the same animals.

| IgG hybridoma | OSE-positive | OSE-negative |
| --- | --- | --- |
| F4_32 | 4.94 |  |
| F6_13 | 1.75 |  |
| F8_07 | 2.11 |  |
| F9_25 | 5.82 |  |
| F9_3_H7 | 1.74 |  |
| F4_61 |  | 1.39 |
| F4_62 |  | 3.09 |
| F6_4 |  | 1.39 |
| F6_8 |  | 0.79 |
| F8_11 |  | 2.72 |
| F9_14 |  | 6.32 |
| F9_21 |  | 1.36 |
| F6_17 |  | 1.04 |
| F7_29 |  | 2.43 |
| F8_6 |  | 3.82 |
| F8_8 |  | 1.36 |
| F9_17 |  | 1.05 |
| F9_13 |  | 7.64 |
| F8_2_H9 |  | 1.74 |
